# Acute effects of caffeine supplementation on taekwondo performance: the influence of competition level and sex

**DOI:** 10.1038/s41598-023-40365-5

**Published:** 2023-08-23

**Authors:** Ibrahim Ouergui, Slaheddine Delleli, Craig Alan Bridge, Hamdi Messaoudi, Hamdi Chtourou, Christopher Garrett Ballmann, Luca Paolo Ardigò, Emerson Franchini

**Affiliations:** 1 High Institute of Sport and Physical Education of Kef, University of Jendouba, 7100 El Kef, Tunisia; 2 Research Unit: Sports Science, Health and Movement, UR22JS01, University of Jendouba, 7100 El Kef, Tunisia; 3 High Institute of Sport and Physical Education of Sfax, University of Sfax, Sfax, Tunisia; 4 Research Unit: Physical Activity, Sport and Health, UR18JS01, National Observatory of Sport, 1003 Tunis, Tunisia; 5 Sports Performance Research Group, Edge Hill University, Wilson Centre, Ormskirk, UK; 6 Department of Human Studies, University of Alabama at Birmingham, 1720 2nd Ave South Birmingham, Birmingham, AL 35294 USA; 7 Department of Teacher Education, NLA University College, Linstows Gate 3, 0166 Oslo, Norway; 8 Martial Arts and Combat Sports Research Group, School of Physical Education and Sport, University of São Paulo, São Paulo, Brazil

**Keywords:** Physiology, Motor control

## Abstract

The purpose of this study was to assess the effects of acute caffeine supplementation on physical performance and perceived exertion during taekwondo-specific tasks in male and female athletes with varying expertise. In a double-blinded, randomized, placebo-controlled crossover study design, 52 young athletes from elite (n = 32; 16 males and 16 females) and sub-elite competitive level (n = 20; 10 males and 10 females) participated. Athletes performed taekwondo-specific tasks including the taekwondo-specific agility test (TSAT), 10 s frequency speed of kick test (FSKT-10 s) and multi-bout FSKT (FSKT-multi) under the following conditions: (1) Caffeine (CAF; 3 mg kg^−1^), placebo (PLA), and no supplement control (CON). Session rating of perceived exertion (s-RPE) was determined after the tests. Findings show that regardless of condition, males performed better than females (*p* < 0.05) and elite athletes had superior performance compared to their sub-elite counterparts (*p* < 0.05). For the TSAT (*p* < 0.001), FSKT-10s (*p* < 0.001), and FSKT-multi (*p* < 0.001), CAF enhanced performance in elite female athletes compared to sub-elite females. Likewise, CAF ingestion resulted in superior performance in elite males compared to sub-elite males for FSKT-10s (*p* = 0.003) and FSKT-multi (*p* < 0.01). The ergogenic potential of CAF during taekwondo-specific tasks appears to be related to a competitive level, with greater benefits in elite than sub-elite athletes.

## Introduction

Caffeine (CAF) is one of the most widely used psychoactive substances in sports and in efforts to increase performance and alertness^1^ with strong evidence to support efficacy and safety^2^. A recent systematic review of CAF supplementation by Grgic et al.^3^ reported that overall, CAF induces benefits in muscular strength, endurance, and anaerobic power. Effective oral dosing of CAF supplementation has been recommended to be between 3 and 6 mg kg^−1^ of body mass, with peak plasma caffeine concentrations occurring ~ 60 min post-ingestion^4^. However, there is significant inter-individual variability in response to CAF supplementation, with many factors mediating athletes’ responsiveness including sex^5,6^, genetic background^7^, and age^8^. Although these moderators’ factors have been investigated, inconsistent effects have been reported^9^, and many of them, such as fitness level, sex, training status, as well as their interactions, remain to be fully elucidated.

Sex-specific differences in responses to CAF supplementation have been widely reported^5,8,10^. For example, Temple et al.^5^ reported that males had greater subjective responses to CAF versus females, but changes in blood pressure were more pronounced in females^5^. Some studies provide evidence to suggest that the ergogenic potential of CAF ingestion on some performance outcomes may differ between males and females, albeit this is still debated^11–13^. Sabblah et al.^12^ reported trends of increased total weight lifted during bench press in males, which was absent in females after CAF ingestion^12^. While the physiological reasoning for these phenomena remains unclear, it has been suggested that the hypoalgesic effects and hemodynamic alterations from CAF consumption may be less pronounced in females versus male counterparts^11,14^.

While several studies have been conducted on a variety of exercise patterns and demographics to identify the optimal CAF dosing regimen for exercise performance (i.e., dose, timing, and form), the influence of training status, competitive level, and sex of athletes have been relatively uncontrolled^1^. Female inclusion in study samples has been reported to be fairly low^15^ with 70% to 100% of primary trials including only males^3^. Equity in this area has been complicated and largely attenuated by the exclusion of the use of oral contraceptives^16^ and differences in menstrual cycle stages^17^. While evidence has suggested that hormonal changes that occur throughout the menstrual cycle can affect the rate of CAF metabolism^1^, both males and females have been reported to experience ergogenic effects with CAF ingestion^13^.

Differences in performance enhancement following CAF ingestion have also been attributed to differences in training status^18^. Boyett et al.^18^ reported greater improvements in cycling time trial performance in trained athletes with CAF ingestion although this effect appears to be diurnal in nature^18^. Furthermore, Collomp et al.^19^ showed that trained swimmers were able to improve swimming sprint velocity with CAF ingestion while untrained counterparts did not experience ergogenic effects. Although unknown at this time, this may in part be explained by differences in muscle-buffering capacity since trained individuals may be able to buffer excess hydrogen ions formed from CAF-induced increases in lactate production and catecholamine release, in concert with increased Ca2^+^ bioavailability^20,21^. Still, others have suggested little to no differences in exercise responses with CAF leaving conclusions from this area convoluted and ambiguous^22^. Thus, more research is needed to determine if training status and other factors interact to modulate athlete responses to CAF, especially in sport-specific contexts.

In combat sports, especially taekwondo, athletes are classed based on their sex, body mass, age, and expertise/competitive level^23^, where commonly the objective for athletes in each category, is to gain a competitive edge. As a widely useful ergogenic aid, CAF has been reported to improve several aspects of performance in combat settings^15,24^, specific tests^25^, and taekwondo simulated combat^26^. During simulated taekwondo combat, caffeine ingestion resulted in increased glycolytic contribution, but the number and time of attacks were not affected^26^. Regarding sex comparison, a recent study investigated the effect of CAF supplementation on physical and psychological aspects in male and female taekwondo athletes and showed that males elicited a higher number of techniques, subjective vitality and feeling scores compared to females after CAF supplementation^25^. While intriguing, few previous studies have specifically addressed the training status and competition level of their athletes which may influence their ability to respond to CAF^15^. Regarding CAF and competitive level in combat sports, only one investigation has been conducted to our knowledge, which has been limited to the sport of boxing, and reported improved performance with CAF regardless of competitive level^27^. However, trained boxers expressed different mood dimensions and greater subjective vitality with CAF ingestion compared to untrained. Given the scant CAF research in combat sports and conflicting evidence about how sex and training level mediate the effects of CAF, the purpose of this study was to determine whether biological sex and competitive level (i.e., elite vs. sub-elite) modulate performance changes with CAF on taekwondo-specific tasks.

We hypothesized that male taekwondo athletes would respond to CAF intake more favourably than females at both elite and sub-elite levels, and the competitive advantage gained through caffeine supplementation would be greater for the elite than for sub-elite athletes.

## Methods

### Participants

A priori power analysis was calculated using the G*Power software (Version 3.1.9.4, University of Kiel, Kiel, Germany) using the F test family (ANOVA: repeated measures, within-between interaction) to determine the required sample size with α set at 0.05 and power (1-β) set at 0.80. The effect size f calculated was at 0.252. The analysis revealed that a total sample size of 40 subjects would be sufficient to find significant differences with an actual power of 0.81. Fifty-two taekwondo athletes (males = 26; females = 26) volunteered to participate in the present study. They were divided into the elite (i.e., competing at the international level) and sub-elite (i.e., competing at regional and national levels), all participating regularly in taekwondo training and not suffering from injuries or medical restrictions during the whole period of experimentation. The characteristics of the participants are presented in Table 1. Habitual CAF consumption was assessed in the present study using the questionnaire of Bühler et al.^28^. Results revealed that athletes had a habitual CAF consumption amount of 1.14 ± 0.49 mg kg^−1^, which is classified as mild caffeine consumers according to Filip et al.^29^. Therefore, 3 mg kg^−1^ of body mass used in the present study has been reported to sufficiently enhance exercise performance in both male and female mild caffeine consumers^4^. Athletes were asked to follow the same diet, avoid strenuous exercises, and refrain from CAF consumption (in drinks and supplements) or any other ergogenic resources during the 48 h before each session. Additionally, athletes were not under any weight loss protocol during the period of experimentation.

**Table 1:** Participants characteristics.

	Elite athletes (n = 32)		Sub-elite athletes (n = 20)	
	Males (n = 16)	Females (n = 16)	Males (n = 10)	Females (n = 10)
Age (years)	18.2 ± 0.8	17.7 ± 0.6	17.7 ± 0.8	17.3 ± 0.5
Height (cm)	182 ± 7	167 ± 10	171 ± 9	164 ± 7
Body mass (kg)	61 ± 9	50 ± 5	61 ± 11	55 ± 8

To minimize the effect of the menstrual cycle on the study outcomes, female athletes were tested during their early follicular phase^30^. The menstrual cycle phase was accurately identified with the help of a period tracker application^31^ using the self-reported onset of menses method. After a detailed explanation of the study’s goals and risks, as well as possible caffeine supplementation side effects, both parents and athletes provided written informed assent and consent, respectively. The study was conducted in accordance with the latest Declaration of Helsinki for human experimentation and was approved by the University of Jendouba Research Ethics Committee (CPP SUD No. 0332/2021) before data collection.

### Data collection

This was a double-blinded, randomized, placebo-controlled crossover study design in which the effect of CAF supplementation was investigated on subsequent taekwondo-specific agility test (TSAT), 10 s frequency speed of kick test (FSKT-10s), multiple frequency speed of kick test (FSKT-multi) performances and the rating of perceived exertion (RPE) in elite and sub-elite male and female taekwondo athletes. After familiarization with the testing procedures (72 h before the experiments), athletes were subjected to 3 different conditions: (1) Control—no treatment (CON), (2) CAF—caffeine ingestion (3 mg kg^−1^ of body mass), (3) weight matched placebo (PLA)—for all-purpose bleached flour. The random assignments of experimental conditions were generated using a Microsoft Office Excel 2007 spreadsheet. For the supplement conditions, each substance was diluted in 200 ml of water. This dose of CAF is within the dose range of efficacy in male taekwondo athletes and has shown minimal to no side effects in this population^25^. Forty-five minutes after supplementation, athletes performed a standardized warm-up session consisting of 10 min of running at 9 km h^−1^ followed by 2 min of taekwondo-specific techniques execution and stretching. Three min of rest after the warm-up, the athletes performed the taekwondo-specific agility test (TSAT)^32^, the 10s and multiple frequency of speed tests (FSKT-10s and FSKT-multi, respectively)^33,34^ after CAF, PLA or CON (i.e., warm-up without any consumption). Thirty min after the completion of the tests, athletes were asked to rate their session rating of perceived exertion (s-RPE) using the scale CR 0–10 adapted by Foster et al.^35^. All sessions were separated by a week washout period. To ensure anonymity, supplements were administered in opaque, unmarked containers and handled by a qualified person. Participants were not allowed to discuss or compare tastes or make assumptions about what they had ingested, supervised by staff to ensure that they drank the full amount and no exchanges were allowed. To verify the blinding success, each athlete was asked to identify what he/she drank, and no participants reported being able to readily discern between the two treatments. All the sessions were conducted at the same time of the day (from 10 a.m. to 12 p.m.) to avoid any diurnal variation in the performance. Athletes were night fasting and then fed.

### Data analysis

#### Taekwondo-specific agility test

The TSAT was completed as previously described^32^. Briefly, the athlete started the test from a guard position with both feet behind the start/finish line. When signalled, he/she moved as quickly as possible to a center point with three sparring partners around them with one partner on each side of them at ~ 180° and the third directly in front of them at ~ 90°. Then, the participant delivered a single roundhouse kick with the dominant leg to each partner on either side and a double roundhouse kick to the third partner as quickly as possible. The athlete then returned to the center point to finish the test^32^. Time to completion was measured using photocells (Brower Timing Systems, Salt Lake City, UT, USA). The intraclass correlation coefficient (ICC) for test–retest in the present study was 0.86.

#### Ten seconds frequency speed of kick test

The athlete must perform the maximum number of kicks against a punching bag by alternating bandal-chagui technique executed by the right and left leg as described by Santos et al.^33^. The number of techniques performed during the 10 s represented the performance during the tests^33^. The ICC for the test–retest in the present study was 0.82.

#### Multiple frequency speed of kick test

The athlete performed five sets of FSKT-10s with a 10-s rest interval between repetitions^34^. Performance was determined by the total number of kicks performed in the 5 sets. ICC for the test–retest in the present study was 0.77.

#### Session rating of perceived exertion

Session rating of perceived exertion was assessed using the 0–10 exertion perception scale adapted by Foster et al.^35^. This is a scale ranging from “0” to “10”, with corresponding verbal expressions, that gradually increases with the intensity of perceived sensation (0 = Rest; 1 = Very Very Easy; 2 = Easy; 3 = Moderate; 4 = Somewhat Hard; 7 = Very Hard; and 10 = Maximal).

### Statistical analysis

The statistical analysis was performed using SPSS 20.0 statistical software (IBM corps., Armonk, NY, USA). Data were presented as mean and standard deviation. The Kolmogorov–Smirnov test was used to check and confirm the normality of data sets, and the Levene test was used to verify the homogeneity of variances. Sphericity was tested using the Mauchly test and a Greenhouse–Geisser correction was used when necessary. A three-way analysis of variance (ANOVA) (condition × group × sex) with repeated measures was used to compare results throughout the different conditions. When significant main effects or interactions were present, a Bonferroni post-hoc test was used to determine mean differences. If an interaction effect was found, this was the sole result reported for a given variable to improve the clarity of the results. For partial eta squared (η_p_^2^) effect size values were reported. Estimates of effect size between mean outcomes used Cohen's d and were interpreted as d ≤ 0.2 (trivial), 0.2 < d ≤ 0.6 (small), 0.6 < d ≤ 1.2 (moderate), 1.2 < d ≤ 2.0 (large), 2.0 < d ≤ 4.0 (very large), and d > 4.0 (extremely large) according to Hopkins^36^. In addition, the upper and lower 95% confidence intervals of the difference (95% CI_diff_) were calculated for the corresponding variation. The level of statistical significance was set at *p* ≤ 0.05.

## Results

### TSAT

There was a significant interaction effect between group and sex (F_1.48_ = 5.065; *p* = 0.029; η_p_^2^ = 0.095; Table 2), with elite females presenting better performances than sub-elite (95% CI_diff_ − 0.8; − 0.3; *p* < 0.001; d = − 1.40), elite males performing better than elite females (95% CI_diff_ − 0.5; − 0.07; *p* = 0.011; d = − 0.85), and sub-elite males presenting better performances than sub-elite females (95% CI_diff_ − 0.9; − 0.4; *p* < 0.001; d = − 1.45). There was a significant interaction effect between group and condition (F_1.58,75.72_ = 3.707; *p* = 0.039; η_p_^2^ = 0.072), with the elite group displaying better performances than sub-elite in CON, CAF and PLA conditions (95% CI_diff_ − 0.4; − 0.01, − 0.7; − 0.3 and − 0.7; − 0.2; *p* = 0.05 and *p* < 0.001; d = − 0.70, − 1.13 and − 0.88, respectively), CAF resulting in better performances than PLA and CON conditions in elite athletes (95% CI_diff_ − 0.6; − 0.4 and − 0.4; − 0.3; both *p* < 0.001; d = − 1.17 and − 1.60, respectively), and PLA condition inducing better performance than CON in elite athletes (95% CI_diff_ − 0.3; − 0.03; *p* = 0.012; d = − 0.55).

**Table 2:** Physical performances and session rating of perceived exertion (s-RPE) recorded during caffeine (CAF), placebo (PLA) and control (CON) conditions in elite and sub-elite male and female taekwondo athletes.

	Elite athletes			Sub-elite athletes		
	M (n = 16)	F (n = 16)	Overall (n = 32)	M (n = 10)	F (n = 10)	Overall (n = 20)
TSAT (s)						
CAF	5.3 ± 0.2*^#†¥^	5.5 ± 0.3*^#¥^	5.4 ± 0.3*^#¥^	5.4 ± 0.5*^#†^	6.3 ± 0.4	5.9 ± 0.6*
PLA	5.6 ± 0 *^†¥^	5.8 ± 0.3*^¥^	5.7 ± 0.3*^¥^	5.7 ± 0.5*^†^	6.6 ± 0.5	6.1 ± 0.7
CON	5.7 ± 0.3^†¥^	6.1 ± 0.3^¥^	5.9 ± 0.4^¥^	6.0 ± 0.2 ^†^	6.3 ± 0.4	6.2 ± 0.4
FSKT-10s (n)						
CAF	28 ± 1*^#†¥^	27 ± 2^#¥^	28 ± 2*^#¥^	27 ± 2*^#†^	24 ± 1	25 ± 2*
PLA	25 ± 1^†¥^	25 ± 1^¥^	25 ± 1^¥^	25 ± 2^†^	23 ± 1	24 ± 2
CON	26 ± 1^†¥^	25 ± 1^¥^	25 ± 1^¥^	24 ± 2^†^	22 ± 1	23 ± 2
FSKT-multi (n)						
CAF	130 ± 1*^†¥^	127 ± 2^¥^	128 ± 2*^¥^	121 ± 8	110 ± 10	115 ± 10*
PLA	125 ± 2*^†¥^	124 ± 1^¥^	124 ± 2^¥^	117 ± 7	105 ± 6	111 ± 9*
CON	125 ± 2^†¥^	120 ± 1^¥^	123 ± 2 ^¥^	107 ± 6	95 ± 9	101 ± 10
s-RPE (a.u.)						
CAF	7.6 ± 1.0	7.8 ± 0.9	7.7 ± 0.9^¥^	7.4 ± 0.8	7.1 ± 1.4	7.2 ± 1.1
PLA	9.4 ± 0.6	8.9 ± 2.2	9.1 ± 1.6^¥^	7.5 ± 1.5	6.9 ± 1.5	7.2 ± 1.5
CON	9.6 ± 0.5	9.3 ± 0.5	9.6 ± 0.5 ^¥^	7.0 ± 1.0	7.7 ± 0.9	7.3 ± 1.0

Values are presented as mean ± SD.

*TSAT* taekwondo agility specific test, *FSKT-10s* ten seconds frequency of speed kick test, *FSKT-multi* multiple frequency of speed kick test, *a.u.* arbitrary unit, *M* male, *F* female.

*Indicates a significantly different from CON (*p* < 0.05).

^#^Indicates significantly different from PLA (*p* < 0.05).

^†^Indicates significantly different from females (*p* < 0.05).

^¥^Indicates significantly different from sub-elite (p < 0.05).

There was a significant interaction effect between sex and condition (F_1.58,75.72_ = 1.236; *p* = 0.289; η_p_^2^ = 0.025), with males showing better performances than females in CON, CAF and PLA conditions (95% CI_diff_ − 0.6; − 0.2, − 0.7; − 0.3 and − 0.8; − 0.3; *p* < 0.001 for all comparisons; d = − 1.27, − 1.05 and − 0.95, respectively). In both males and females, CAF resulted in better performances than CON (95% CI_diff_ − 0.6; − 0.3 and − 0.5; − 0.3; both *p* < 0.001; d = − 1.31 and − 0.92, respectively) and PLA (95% CI_diff_ both − 0.4; − 0.2; both *p* < 0.001; d = − 0.87 and − 0.56, respectively) conditions.

There was a significant interaction effect between group, sex, and condition (F_1.58,75.72_ = 15.702; *p* < 0.001; η_p_^2^ = 0.246). In elite male athletes, CAF resulted in better performances than CON and PLA conditions (95% CI_diff_ − 0.5; − 0.2 and − 0.2; − 0.3; both *p* < 0.001; d = − 1.36 and − 1.48, respectively), as well as compared with females (95% CI_diff_ − 0.8; − 0.5 and − 0.4; − 0.2; both *p* < 0.001; d = − 2.45 and − 1.04, respectively), and for elite females with PLA compared to the CON condition (95% CI_diff_ − 0.5; − 0.1; *p* = 0.001; d = − 1.13). Similarly, sub-elite male athletes with CAF presented better performances than CON and PLA conditions (95% CI_diff_ − 0.7; − 0.3 and − 0.4; − 0.1; *p* < 0.001 and p = 0.004; d = − 1.46 and − 0.47, respectively), and with PLA compared to the CON condition (95% CI_diff_ − 0.6; − 0.02; *p* = 0.033; d = − 0.83). Likewise, sub-elite female athletes with CAF performed better than in PLA condition (95% CI_diff_ − 0.5; − 0.1; *p* = 0.001; d = − 0.72). Additionally, elite male athletes elicited better performances than elite females in CON condition (95% CI_diff_ − 0.7; − 0.02; *p* < 0.001; d = − 1.62), and sub-elite male athletes performed better than sub-elite females in CON, CAF and PLA conditions (95% CI_diff_ − 0.6; − 0.1, − 1.1; − 0.5 and − 1.2; − 0.5; *p* = 0.017 and *p* < 0.001; d = − 0.96, − 1.85 and − 1.68, respectively). Furthermore, elite male athletes presented better performances than sub-elite males in the CON condition (95% CI_diff_ − 0.6; − 0.1; *p* = 0.014; d = − 1.14), while elite female athletes performed better than sub-elite female athletes in CAF and PLA conditions (95% CI_diff_ − 1.1; − 0.5 and − 1.1; − 0.4; both *p* < 0.001; d = − 2.36 and − 1.89, respectively).

### FSKT-10s

There was a significant interaction effect between group and sex (F_1.48_ = 4.469; *p* = 0.040; η_p_^2^ = 0.085), with elite male athletes eliciting better performance than sub-elite males (95% CI_diff_ 0.2; 1.9; *p* = 0.016; d = 0.58), and elite females presenting higher performance than sub-elite females (95% CI_diff_ 1.5; 3.2; *p* < 0.001; d = 1.57). In addition, elite males’ performance was higher than elite females (95% CI_diff_ 0.5; 2.0; *p* = 0.002; d = 0.76), and sub-elite males performed better than sub-elite females (95% CI_diff_ 1.6; 3.5; *p* < 0.001; d = 1.46). Similarly, there was a significant interaction effect between condition and group (F_2.96_ = 4.319; *p* = 0.016; η_p_^2^ = 0.082), with elite athletes performing better than sub-elite in CON, CAF and PLA conditions (95% CI_diff_ 1.0; 2.4, 1.5; 3.1 and 0.3; 2.2; both *p* < 0.001 and *p* = 0.006; d = 1.17, 1.28 and 0.70, respectively). Likewise, CAF induced better performances than CON and PLA conditions with elite (95% CI_diff_ 1.9; 3.2 and 1.7; 2.8; both *p* < 0.001; d = 1.73 and 1.66, respectively) and sub-elite (95% CI_diff_ 1.0; 2.7 and 0.4; 1.8; *p* < 0.001 and *p* = 0.001; d = 1.00 and 0.49, respectively) athletes.

There was a significant interaction effect between condition, group and sex (F_2.96_ = 3.953; *p* = 0.022; η_p_^2^ = 0.076), with elite male and female athletes performing better with CAF compared to CON (95% CI_diff_ 1.7; 3.7 and 1.4; 3.3; both *p* < 0.001; d = 2.88 and 0.95, respectively) and PLA (95% CI_diff_ 2.2; 3.6 and 0.8; 2.3; both *p* < 0.001; d = 2.91 and 1.14, respectively) conditions, and sub-elite male athletes with CAF compared to CON and PLA conditions (95% CI_diff_ 1.4; 3.8 and 0.2; 2.2; *p* = 0.009 and *p* < 0.001; d = 1.51 and 0.59, respectively), while female sub-elite athletes induced better performance with CAF compared to PLA (95% CI_diff_ 0.04; 1.9; *p* = 0.038; d = 0.79). Moreover, elite males performed better than females in CON (95% CI_diff_ 0.6; 2.4; *p* = 0.001; d = 1.33) and CAF (95% CI_diff_ 0.9; 2.9; *p* < 0.001; d = 1.43) conditions, and sub-elite males elicited higher performance than females in CON (95% CI_diff_ 0.6; 2.4; *p* = 0.003; d = 1.25), CAF (95% CI_diff_ 1.8; 4.4; *p* < 0.001; d = 2.0) and PLA (95% CI_diff_ 1.6; 4.2; *p* < 0.001; d = 2.02) conditions. In addition, elite male athletes elicited better performance than sub-elite in CON and CAF conditions (95% CI_diff_ 0.6; 2.6 and 0.6; 2.8; *p* = 0.002 and *p* = 0.003; d = 1.30 and 1.28, respectively), and elite females performed better than sub-elite females in CON, CAF and PLA conditions (95% CI_diff_ 0.8; 2.8, 1.8; 4.1 and 1.2; 3.5; *p* = 0.001 and both *p* < 0.001; d = 1.48, 2.04 and 2.01, respectively).

### FSKT-multi

There was a significant interaction effect between condition and group (F_1.47.70.80_ = 18.548; *p* < 0.001; η_p_^2^ = 0.221), with elite athletes eliciting better performance than sub-elite in CON, CAF and PLA conditions (95% CI_diff_ 19.3; 25.3, 9.3; 15.9 and 11.3; 16.0; *p* < 0.001 for all comparisons; d = 3.49, 1.90 and 2.46, respectively), and elite athletes performing better in CAF compared to CON and PLA conditions (95% CI_diff_ 1.5; 8.2 and 1.8; 5.7; both *p* < 0.001; d = 2.50 and 2.08, respectively), as well as compared with sub-elite athletes (95% CI_diff_ 10.3; 18.7 and 2.3; 7.2; both *p* < 0.001; d = 1.41 and 0.50, respectively). There was a significant interaction effect between group and sex (F_1.48_ = 20.622; *p* < 0.001; η_p_^2^ = 0.300), with elite eliciting better performance than sub-elite athletes for males (95% CI_diff_ 8.6; 14.4; *p* < 0.001; d = 1.91) and females (95% CI_diff_ 17.9; 23.8; *p* < 0.001; d = 3.10), and elite males resulting in better performance than elite females (95% CI_diff_ 8.3; 14.8; *p* < 0.001; d = 0.82).

### s-RPE

A significant interaction effect between condition and group was found (F_1.74.83.34_ = 10.261; *p* < 0.001; η_p_^2^ = 0.176), with elite group reporting higher values than sub-elite athletes in CON and PLA conditions (95% CI_diff_ 1.8; 2.7 and 1.1; 2.8; both *p* < 0.001; d = 2.98 and 1.26, respectively), and elite group reporting lower values with CAF than CON and PLA conditions (95% CI_diff_ − 2.4; − 1.4 and − 2.2; − 0.8; both *p* < 0.001; d = − 2.54 and − 1.13, respectively).

All raw results are reported in Tables S1, S2 and S3.

## Discussion

This study investigated whether the competitive level and sex may influence the acute effects of CAF intake on taekwondo-specific performances. The main findings suggest that elite and sub-elite athletes of both sexes experienced ergogenic benefits from CAF when compared to PLA and CON conditions and that males performed better compared to females regardless of competitive level and/or condition. In addition, males performed better in agility compared to females in PLA and CAF conditions. Even though the distinct mechanisms underpinning these results are not fully known, data suggest that performance enhancement during taekwondo with CAF ingestion is intimately dependent on the competitive level mainly for female athletes. Specifically, while the magnitude of performance improvement from CAF intake was comparable across elite and sub-elite male athletes (difference only in FSKT-10s), elite female athletes showed greater magnitudes of gains from CAF intake than sub-elite females for both TSAT and FSKT-10s. As well, elite and sub-elite males with CAF elicited better performance than elite and sub-elite females for TSAT and FSKT-10s.

The results of the present study showing improved agility and kicking performance with CAF ingestion are comparable to those previously reported in other investigations on combat sports^25,37^. The performance improvement following CAF supplementation may be explained by numerous physiological mechanisms since CAF may increase motor unit recruitment, motor unit synchronization, muscle fiber conduction velocity, and enhance voluntary activation^38^. Furthermore, intra and inter-muscular coordination is enhanced with CAF ingestion^39^. Thus, these physiological aspects related to CAF intake might result in more forceful muscle contractions, reinforcing the notion that CAF may be used to enhance muscle power production and movement velocity^40^.

The present study provides novel insights into how CAF influences exercise performance related to the competitive level of female athletes. Due to the influence of body composition, aerobic capacity, and some anaerobic measures that are mediated by hormonal fluctuations, sex has been recognized as a significant factor in determining athletic performance^41^. In previous studies, caffeine appeared to induce a higher ergogenic effect in males during anaerobic exercises^6^. Particularly, with the same dose of CAF, males presented greater ergogenic effects than females, mainly in strength and speed tasks^42^. Although previous research indicated that female athletes benefit from CAF intake throughout the menstrual cycle^43^, it is still likely that females obtain lower ergogenic effects from oral CAF intake than males due to the interaction of CAF and female sex hormones^4^. In both elite and sub-elite levels, the results of the current study showed that females performed worse than males even with CAF ingestion. This could be unpinned by the speed of CAF metabolism which may be a potential factor explaining how males and females differ^1^. It has been shown that metabolic enzymes (CYP1A2 and ADORA2A) regulating the rate of CAF metabolism, are linked to genotypic features of the athlete and result in various responses to CAF^7^. Even when sharing similar genotypes in this regard, females metabolize CAF more slowly than males^1^. Reasonably, the fluctuations in female sex hormones during the menstrual cycle might restrain the metabolism of caffeine and thus alter the efficacy of this supplement to improve performance^44^. As a result, females benefit from caffeine intake following a long interval between the time of administration and the subsequent task^45^. In addition, considering that CAF benefits are greater in individuals with larger muscle mass^46^, and given that males tend to possess greater muscle mass than females, this may partially explain greater performances recorded in males than females^47^. From a hormonal approach, CAF supplementation may increase circulating testosterone in response to exercise^48^, which may increase risk-taking behaviors and aggression^49^, which are required reactions in combat sports^15^. However, the acute effects and interactions of CAF and testosterone in combat sports have yet to be studied and should be the subject of future investigations.

Sex has also been suggested to moderate the link between CAF supplementation and arousal alterations^50^. Specifically, males appear to show less somnolence and more arousal following CAF supplementation when compared to females^50^. This may indicate greater antagonism of adenosine receptors by CAF in males which would support current findings of larger magnitudes of agility performance enhancement with CAF in males compared to female taekwondo athletes. It is plausible that CAF antagonism of adenosine receptors increases locomotor activity in males more than females^11^. In this consideration, Chen et al.^11^ showed that after CAF intake, male athletes experienced a greater reduction in delayed-onset muscle soreness with the better restoration of impaired maximal voluntary isometric contractions than females. Additionally, Ouergui et al.^25^ showed that more positive affect and vitality profiles were recorded in males than females after CAF intake.

Regarding the competitive level, due to the high performance and capabilities of highly trained individuals, it was questioned if performance can be further enhanced following CAF ingestion^1^. This was based on the fact that in highly trained individuals (i.e., elite athletes), there is less improvement following CAF ingestion, as these individuals are, by definition, towards the upper limit of human exercise performance capabilities and are approaching absolute physical limits^51^, i.e., a ceiling effect is likely to occur. However, the present study confirmed that despite their high performance level, elite female athletes benefited more from CAF supplementation. Compared to control and placebo conditions, the magnitude of performance improvement in FSKT-10s and FSKT-multi in response to CAF intake differed between elite and sub-elite male athletes (only agility performance was similar), as well as between elite and sub-elite female athletes. Studies comparing combat sports athletes from different competitive levels are lacking and only the study of Jodra et al.^27^ has investigated this and did not report differences between elite and recreationally trained male boxers in terms of anaerobic performance (i.e., peak and mean power) following CAF supplementation of 5 mg kg^−1^. While the results of the present study confirm partially those of Jodra et al.^27^, the improvement during the kicking performance tests (FSKT-10s and FSKT-multi) in favor of elite male athletes may manifest in the type of exercise/task. In fact, with a lower amount of CAF intake during our study as compared to the procedure of Jodra et al.^27^, this difference might be attributed, in addition to the inter-individual variability^9^, to the type of tests used in both studies. Jodra et al.^27^ used a standard 30 s Wingate cycling test to assess anaerobic capacity in elite and recreationally trained male boxers, which lacks mechanical specificity to adaptations in the boxing group. This may well have masked any detectable differences in the ergogenicity of CAF between the groups. In contrast, taekwondo-specific exercise tests were implemented in the current study and both groups were actively training in taekwondo. This may highlight the importance of employing more valid and specific tests to enhance sensitivity in detecting differences in the ergogenic action of caffeine between standards of competitors^27^. It is pertinent to note, however, that, unlike performance on FSKT-10s and FSKT-multi, performance on the agility tests following caffeine ingestion was comparable between competition levels. While it is not possible to fully elucidate the cause of these incongruent responses, it could be a function of differences in the energetic demands of the tests, or variation in the test’s validity, reliability, and/or sensitivity. Future research might want to consider the importance of these aspects.

The ergogenic effects of caffeine on exercise performance appear to be predominantly related to CAF’s binding to adenosine receptors^52^. The current investigation showed that taking CAF effectively reduced s-RPE within elite male athletes compared to placebo and control conditions. Similarly, Jodra et al.^27^ reported that elite athletes have benefited more from CAF supplementation in RPE regarding both muscular pain felt at legs and cardiorespiratory level compared to the placebo condition. However, Silva-Lopes et al.^26^ did not report any difference in RPE after each round of a simulated taekwondo match in CAF or PLA conditions. Saldanha da Silva Athayde et al.^53^ also did not report any difference in RPE values recorded after successive judo matches between caffeine and placebo conditions. In the present study, the improvement in physical performance within elite male subgroup was accompanied by s-RPE decrease, which may indicate that among the ergogenic effects of CAF is the diminished sensation of fatigue induced by exercise^54^. This could potentially have favorable effects on negating the decreased firing rates of motor units and possibly producing a more sustainable and forceful muscle contraction^55^.

The main strength of the current study was that we employed a double-blind, randomized, placebo-controlled crossover study design to examine the influence of caffeine on athletes’ performance using sports-specific tests (reflecting the mechanical actions, activity patterns and metabolic demands of the sport), and in relation to sex and competition level. Nevertheless, several aspects require further consideration. While the results demonstrate the ergogenic potential of caffeine on performance in taekwondo-specific tests, and differences in its ergogenicity between competitive levels and sexes, its translation to performance in competition or simulated competition remains to be fully elucidated. Furthermore, we did not attempt to quantify the physiological mechanisms underlying caffeine’s performance improvement or variation in the responses between competitive level and sex. Differences in body mass and body composition between sexes could mean that females received a more potent dose of caffeine than males^42^, and females would take longer to metabolize caffeine^1^. In addition, in combat sports the ergogenic potential of CAF on performances was generally accompanied by an increased glycolytic anaerobic metabolism^15,26^. In this context, Skinner et al.^56^ explained that, regardless of dosage, the occurrence of CAF’s ergogenic benefit at the peripheral level requires a greater availability of the substance in the muscle (i.e., increased concentrations of CAF at the site of action). Although the performance enhancement in the present study could be attributed to the increase in CAF bioavailability, the rate of its metabolism could vary across participants based on their sex and genetic background^5–7^. However, since objective markers such as urine CAF production, plasma CAF, or CAF metabolite concentrations were not measured, the bioavailability of CAF can be considered a limitation. In the present study, we used a single dose and standardized rest interval before exercising. Finally, for some variables, the data demonstrated significant differences in performance between placebo and control conditions, as well as between caffeine and control, and caffeine and placebo. The former suggests that the process of consuming something that is believed to be ergogenic may have had a small positive impact on performance (small effect size) in this study (a placebo effect)^57^. Nevertheless, the observation of significant differences between caffeine and placebo conditions would confer the ergogenic potential of caffeine in this setting, above and beyond any placebo effect. The potential ‘additive effects’ of placebo and caffeine on performance outcomes cannot therefore be discounted in this setting and offers an interesting area for future study.

## Conclusions

The present study showed that low doses of caffeine ingestion improved both agility and kicking performances of young taekwondo athletes. Moreover, the ergogenic potential of CAF appeared to be related to sex and competitive level, with greater benefits in elite than sub-elite for females and males (only kicking performance tests) and in males compared with females for both elite and sub-elite levels. In addition, caffeine supplementation was able to reduce s-RPE more than CON and PLA conditions in elite athletes. These findings suggest that low-dose CAF supplementation may be used by both male and female athletes from different competitive levels to optimize training and cope with the demands of taekwondo. Future studies are warranted before true recommendations can be made for taekwondo athletes regarding the use of CAF.

### Supplementary Information

Supplementary Tables.

## Data Availability

All data generated and analysed during this study are included in this published article and its supplementary information file.
